# Current Concepts in Intra-articular Calcaneus Fractures

**DOI:** 10.1055/s-0045-1809886

**Published:** 2025-07-29

**Authors:** Rafael Barban Sposeto, Germán Matías-Joannas, Alexandre Leme Godoy-Santos

**Affiliations:** 1 Department of Orthopedics and Traumatology, Instituto de Ortopedia, Hospital das Clínicas da Universidade de São Paulo, São Paulo, SP, Brazil; 2 Foot and Ankle Division, Centro de Estudios y Práctica Profesional (CEPP), Instituto Dupuytren, Buenos Aires, Argentina

**Keywords:** calcaneus, fracture fixation, internal, fractures, bone, intra-articular fractures, subtalar joint, tarsal bones, articulação talocalcânea, calcâneo, fixação interna de fraturas, fraturas intra-articulares, fraturas ósseas, ossos do tarso

## Abstract

Intra-articular calcaneal fractures are significant injuries to the locomotor system, often leading to lifelong impairments in foot mechanics with substantial occupational, social, and financial repercussions. The initial imaging diagnosis relies on radiography; however, computed tomography is crucial for understanding the three-dimensional anatomy of the fracture and facilitating surgical planning.

The treatment of intra-articular calcaneal fractures remains a subject of debate, with literature supporting diverse approaches for similar fracture types. Currently, the gold standard for managing subtalar joint fractures is surgical intervention. Among the two most common techniques, the extended lateral and the sinus tarsi approaches yield comparable functional outcomes, though the latter is associated with fewer complications.

The present article discusses the diagnosis, classification, and treatment of intra-articular calcaneal fractures, focusing on the sinus tarsi and extended lateral “L” approaches, as well as the fixation techniques applicable to each fracture type.

## Resumo

As fraturas intra-articulares do calcâneo são lesões significativas do sistema locomotor, muitas vezes levando a deficiências permanentes na mecânica do pé, com repercussões ocupacionais, sociais e financeiras importantes. A primeira técnica de diagnóstico por imagem é a radiografia; no entanto, a tomografia computadorizada é crucial para entender a anatomia tridimensional da fratura e facilitar o planejamento cirúrgico.

O tratamento de fraturas intra-articulares do calcâneo ainda é debatido, e a literatura apoia diversas abordagens para tipos de fraturas semelhantes. Hoje, o padrão-ouro para o tratamento de fraturas da articulação subtalar é a intervenção cirúrgica. Entre as duas técnicas mais comuns, a abordagem lateral estendida e a abordagem do seio do tarso produzem desfechos funcionais comparáveis, embora a última esteja associada a menos complicações.

Este artigo discute o diagnóstico, a classificação e o tratamento das fraturas intra-articulares do calcâneo, com foco nas abordagens do seio do tarso e do “L” lateral estendido, bem como as técnicas de fixação aplicáveis a cada tipo de fratura.

## Introduction

The calcaneus is the largest tarsal bone. Among foot bones, the calcaneus is the most frequently injured (60%), accounting for 1 to 2% of all fractures in the body. Additionally, 75% of them are intra-articular fractures. 1 2 These are some of the most challenging articular fractures to manage, often yielding unsatisfactory outcomes for both patients and physicians. 3

Most calcaneal fractures result from high-energy trauma and primarily occur in young, active patients. 4

There is controversy regarding the optimal treatment for these fractures. Both surgical and non-surgical approaches have their advantages and disadvantages. However, over time, surgical management has emerged as the preferred approach for calcaneal fractures. 2 4 5 6 7

The literature demonstrates that anatomical reduction and internal fixation provide the best outcomes in terms of rapid recovery and early restoration of subtalar joint function. 7 8 The gold standard in treatment should include anatomical reduction of the subtalar joint, restoration of the normal width, alignment, and length of the calcaneus, and stabilization with rigid fixation. 5

## Anatomy

The calcaneus, along with the talus, forms the hindfoot. It features four articular surfaces: a calcaneocuboid and a subtalar joint, which is further divided into anterior, middle, and posterior facets. The posterior subtalar facet is the largest and most critical for load support during gait, presenting a convex shape oriented distally and laterally at a 45° angle to the sagittal plane. 9 The middle facet is located on the upper surface of the sustentaculum tali, anterior and medial to the posterior facet. The anterior facet, which is the smallest one, is found on the anterior aspect of the calcaneus, situated laterally to the sustentaculum tali. 9 10

The medial surface of the calcaneus contains a thick cortex that supports its medial projection, the sustentaculum tali. This structure provides stability to the head and neck of the talus, playing a key role in maintaining hindfoot alignment. It serves as the insertion site for the tibiocalcaneal component of the deltoid and the spring ligaments. 10

## Trauma Mechanism

Although calcaneal fractures can result from pathological conditions such as tumors or stress injuries, most cases are caused by high-energy axial trauma affecting the calcaneal joints. 2 11 12

Intra-articular calcaneal fractures arise from axial forces that disrupt the subtalar and calcaneocuboid joints, as well as the calcaneal body and surrounding soft tissues. 4 13 The resulting fracture pattern depends on the direction and intensity of the force, the position of the foot at the time of injury, and the patient's bone quality. 1

Axial forces compress the calcaneus against the talus, with the lateral talar process impacting the calcaneal cortex at the Gissane angle. Because the talus's load axis is more medial than that of the calcaneus, an eversion moment occurs, producing the primary fracture line. 1

This primary fracture line divides the calcaneus coronally into an anteromedial and a posterolateral fragment. The anteromedial fragment, which includes the sustentaculum tali and the middle subtalar facet, remains congruent with the talus. Due to its consistent anatomical relationship, this fragment—termed the “constant fragment”—is an important reference for reduction. 2 14

Hindfoot valgus at the time of trauma results in a more lateral primary line and a larger anteromedial fragment, whereas hindfoot varus generates a more medial line and a smaller fragment, complicating reduction and fixation during surgery. 1

As trauma energy dissipates, secondary fracture lines create comminution across the calcaneal body and the subtalar and calcaneocuboid joints. 7 14 15 Typical deformities include varus alignment of the calcaneal tuberosity, depression of the posterior subtalar joint, and lateral wall widening, with proximal displacement of the tuberosity and reductions in both calcaneal pitch and Böhler's angle.

The resulting tuberosity varus is a composition of movement of this segment in the three planes. Lateral translation of calcaneal tuberosity in conjunction with medial rotational movement results in varus of the coronal axis of the tuberosity. 1

## Physical Exam

Calcaneal fractures are often associated with injuries to other musculoskeletal regions and body systems. A comprehensive evaluation of patients, following Advanced Trauma Life Support (ATLS) protocols, is essential. Commonly associated injuries include fractures of the tibial plafond, tibial plateau, and spine. 4

During the foot examination, it is crucial to assess pain and deformities in the hindfoot, midfoot, and forefoot, along with skin lesions, sensory deficits, pulses, and perfusion. Diagnosing compartment syndrome at this stage is vital, as it manifests as tense edema, pain unresponsive to potent analgesics, severe pain during toe movement, perfusion deficits, and paresthesia. 2

Common clinical findings in calcaneal fractures include plantar ecchymosis, pain, edema, hindfoot deformities, and difficulty bearing weight. Soft-tissue integrity is critical in determining surgical timing and planning. The presence of phlyctenae often indicates delayed surgical intervention. Surgery is typically scheduled after edema reduction, when skin wrinkling and phlyctena healing are observed. 1

## Diagnostic Imaging Techniques

### Radiographic Evaluation

Radiography is the primary imaging modality for diagnosing calcaneal fractures and should include lateral, anteroposterior, and oblique foot views, along with axial views of the calcaneus. 16

**Fig. 1:**
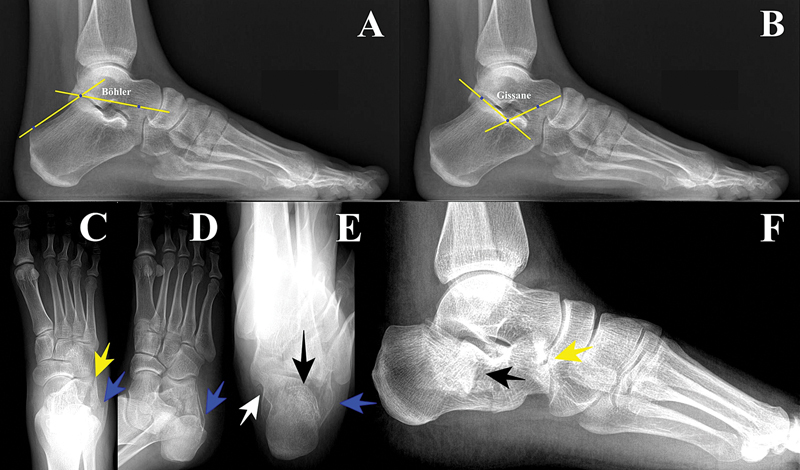
Radiographic evaluation. ( **A** ) Böhler angle in normal foot (values range from 20–40°). It is formed by the intersection of lines connecting the anterior process, the posterior subtalar joint, and the calcaneal tuberosity. ( **B** ) Gissane's angle in normal foot (values between 95–105°). It is formed by the line of the posterior facet and the line from the sulcus to the most superior portion of the anterior calcaneal process. ( **C** ) Anteroposterior incidence, with calcaneal fracture. ( **D** ) Oblique incidence, with calcaneal fracture. ( **E** ) Axial incidence, with calcaneal fracture. ( **F** ) Lateral incidence, with calcaneal fracture. Yellow arrow – calcaneocuboid articular fracture. Blue arrow – Lateral wall enlargement. Black arrow – Posterior subtalar joint depression. White arrow – Constant fragment.

Lateral radiographs are used to measure Böhler's and Gissane's angles. Böhler's angle is formed by the intersection of lines connecting the anterior process, the posterior subtalar joint, and the calcaneal tuberosity. Normal values range from 20 to 40°. 17 Gissane's angle, measured from the lateral calcaneal wall, lies between 95° and 105° 18 ( Fig. 1 ).

The anteroposterior view better visualizes the calcaneocuboid joint and lateral wall widening. Oblique views show the calcaneocuboid joint and tuberosity displacement relative to the lateral wall. Axial radiographs reveal lateral-medial widening, varus or valgus deviation, and posterior subtalar joint misalignment ( Fig. 1 ).

The adjustment during the reduction of the Böhler's and Gissane's angles, especially the Böhler, restores the shape of the calcaneus body and hindfoot, providing better functional results. 4 12 17

The Broden incidence complements radiographic evaluation. 4 With the patient supine and the ankle in a neutral position, the leg is internally rotated by 30 to 40°. Images are taken at cephalad angles of 40°, 30°, 20°, and 10°, centered on the lateral malleolus, providing detailed visualization of the posterior subtalar joint. Intraoperatively, these views are particularly useful for assessing joint reduction. 4 19

### Computed Tomography

**Fig. 2:**
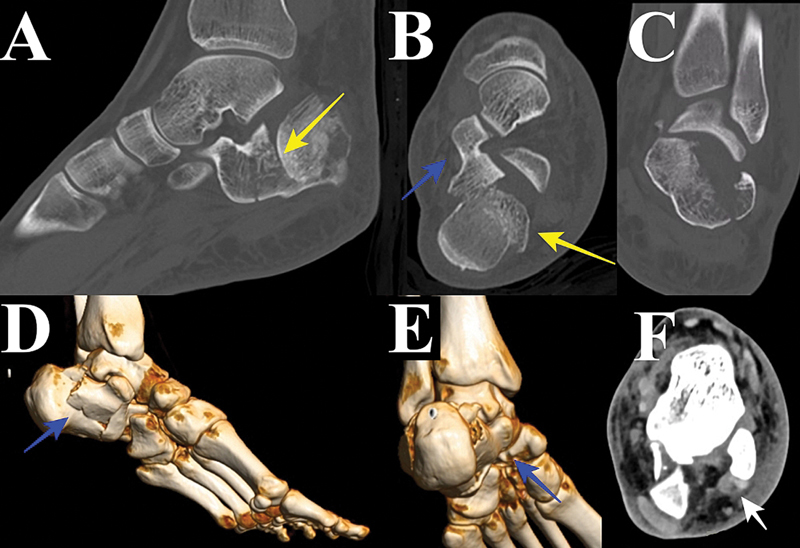
Tomographic evaluation. ( **A** ) Sagittal computed tomography (CT). Subtalar joint depression. ( **B** ) Axial CT. Blue arrow – constant fragment; Yellow arrow – subtalar joint depression. ( **C** ) Coronal CT. Lateral wall enlargement and subtalar articulation incongruence. ( **D** ) 3D CT reconstruction. Blue arrow – constant fragment. ( **E** ) 3D CT reconstruction. Blue arrow – constant fragment. Note rotational displacement of the tuberosity, with lateral translation. ( **F** ) Axial soft tissue CT window. White arrow – peroneal tendons.

Computed tomography (CT) enhances fracture diagnosis, classification, and prognosis, 3 14 15 particularly in identifying associated injuries and understanding three-dimensional fracture displacement for surgical planning 16 20 ( Fig. 2 ).

As CT is a submilimetrical sectional study, it provides better visualization of the articular displacement, assisting in the treatment decision. Furthermore, the CT reconstructions allow better comprehension of the fracture anatomy, helping with the surgical planning, with possible reductions maneuvers and fixation choices.

### Magnetic Resonance Imaging

Magnetic resonance imaging (MRI), though less commonly available and more expensive, is valuable for diagnosing occult and pathological fractures (e.g., tumors, stress fractures), as well as those associated soft-tissue injuries. 21

## Classification

Several classifications have been described for calcaneal fractures.

Böhler was the first to present an extensive classification of calcaneal fractures.

In 1952, Essex-Lopresti described a new system classifying calcaneal fractures into two groups based on the fracture mechanism: tongue-type and joint-depression fractures. 22

In 1989, Rammelt and Zwipp 1 introduced a 12-point classification system for calcaneal fractures, incorporating factors such as the number of joint surfaces involved, main fracture fragments, extent of soft-tissue trauma, and associated fractures of neighboring bones.

In 1993, Sanders et al. 3 published a series of 132 displaced calcaneal fractures, proposing a CT-based classification system. This system is based on coronal sections in an oblique direction, evaluating the posterior facet and talar support in the same section, identifying three fracture lines, A, B and C (from lateral to medial).

- Type-1 fractures do not show displacement, regardless of the number of fracture lines.
- Type-2 fractures involve 2 fragments (a single fracture line) and are further categorized as 2a, 2b, or 2c, depending on the primary fracture line's location.
- Type-3 fractures involve 3 fragments (2 fracture lines) with a depressed central fragment and are similarly classified into 3ab, 3bc, or 3ac.
- Type-4 fractures involve 4 or more fragments with significant comminution. 3

## Treatment of Calcaneal Articular Fractures

In general, surgical treatment provides superior functional outcomes in displaced subtalar joint fractures, regardless of the classification. 23 24 In cases of posttraumatic subtalar osteoarthritis, the outcomes of arthrodesis are better if the acute fracture is initially treated surgically since the alignment of the calcaneal body and the joint has been previously reestablished. 24

Special considerations, such as vascular diseases, smoking, diabetes, advanced age, systemic illnesses, borderline clinical conditions, and extensive soft-tissue injuries, must guide surgical decision-making. Surgical treatment is indicated when the posterior subtalar joint deviation exceeds 2 mm, as defined by Sanders. 3 14 15

Following the AO principles ( *Arbeitsgemeinschaft für Osteosynthesefragen* , AO, in German), anatomical reduction and absolute stability should be prioritized for articular regions. 25 Direct visualization of articular components during open reduction is the preferred method to achieve these goals.

Aligning the calcaneal body is critical to restoring functional relationships within the hindfoot and midfoot. Surgical objectives include correcting Böhler's angle, calcaneal height, length, and morphology. 17 26 In the sagittal plane, posterior and plantar repositioning of the tuberosity are performed, while in the coronal plane, varus alignment, tuberosity translation, and lateral wall widening are corrected.

The reduction of the calcaneal body must realign the fracture components with an indirect functional reduction, which can be achieved by closed means. Relative stability is adequate for body fixation.

In most cases, lateral approaches are used for this purpose. The lateral “L” extended approach allows for open reduction of the subtalar and calcaneocuboid joints and the body of the calcaneus, whereas the minimally invasive approach, the sinus tarsi approach, allows for open reduction of the subtalar and calcaneocuboid joints and closed reduction of the body.

Prather et al., 27 in an experimental study on cadavers, compared the extended approach with the sinus tarsi and demonstrated that both approaches provide equivalent joint exposure area, although the extended approach provides better visualization of the lateral wall.

## Surgical Approach

### Sinus Tarsi

The sinus tarsi approach minimizes damage to surrounding soft tissues, reducing the risk of dehiscence and infection. 28 29 30

Yao et al. 30 in a systematic review with a meta-analysis of 12 studies, compared wound complications and quality of reduction between patients with calcaneal fractures treated with sinus tarsi and extended approaches. The authors demonstrated that the sinus tarsi group had a lower incidence of wound complications, with comparable quality of reduction.

This approach provides reductions comparable to the extended lateral “L” approach, with similar functional outcomes but fewer complications. However, the sinus tarsi technique is technically demanding and requires a longer learning curve. 5 31 32 33 34

With the patient in the lateral decubitus position, the incision begins posterior to the lateral malleolus and extends toward the base of the fourth metatarsal. The length depends on fracture involvement of the calcaneocuboid joint. Subcutaneous dissection reaches the extensor digitorum brevis muscle, which is reflected distally to expose the anterior calcaneus. Care must be taken with the sensitive branches of the sural nerve.

Dissection at the Gissane angle exposes the sinus tarsi, allowing visualization of the interosseous ligaments and posterior subtalar joint. Fibular tendons are moved posteriorly to increase exposure. If necessary, fibulocalcaneal and subtalar interosseous ligaments are released.

**Fig. 3:**
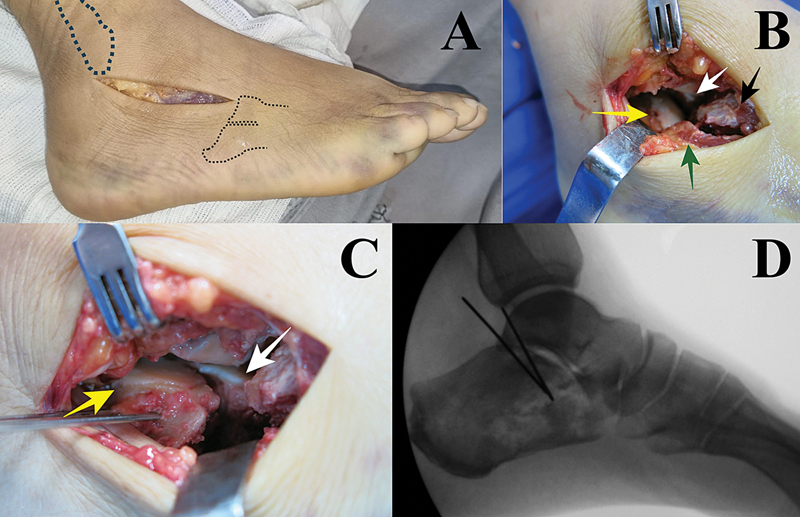
Sinus tarsi approach. ( **A** ) Incision parameters. ( **B** ) Fragments visualization through the incision. Yellow arrow – subtalar joint depression; White arrow – constant fragment; Black arrow – calcaneal anterior portion; Green arrow – lateral wall. ( **C** ) Subtalar joint direct reduction. Yellow arrow – subtalar joint depression; White arrow – constant fragment. ( **D** ) Temporary fixation with Kirshner wires.

Plantar to the lower edge of the incision, under the fibular tendons, with blunt dissection, we gain access to the lateral wall of the calcaneus and, posteriorly, to the tuberosity. Therefore, the sinus tarsi approach allows direct visualization of the posterior subtalar and calcaneocuboid joints, the upper part of the lateral wall of the calcaneus, and the fibular tendons ( Fig. 3 ).

The Essex-Lopresti maneuver, 22 visualized through the sinus tarsi approach, is particularly effective for tongue fractures. In these cases, the fractured lateral fragment of the posterior subtalar joint remains attached to the tuberosity. The goal of the maneuver is to elevate the articular fragment by pushing the tuberosity distally. The sinus tarsi approach enables direct visualization of the anatomical reduction of the lateral subtalar fragment in alignment with the medial constant fragment.

The procedure begins by plantarflexing the ankle and using a Steinmann wire or Schanz pin to push the calcaneal tuberosity distally. Blunt instruments assist in achieving a precise reduction of the joint. Once reduction is achieved, temporary fixation with Kirschner wires ensures the anatomical alignment of the joint and the functional reduction of the calcaneal body. Definitive fixation is then performed using traction screws for the subtalar joint and positioning screws for the tuberosity.

Depression fractures, which are more comminuted and challenging to reduce, require the release of bone fragments from the joint and calcaneal body to create space for alignment. 2 Reduction begins with manipulation of the anterior calcaneus to restore the calcaneocuboid joint, establishing an additional parameter for reduction with the sustentaculum tali.

Fragments of the posterior subtalar joint are then elevated proximally using blunt instruments, aligning them with the medial joint remnants at the sustentaculum tali. The tuberosity is repositioned by introducing a blunt instrument laterally beneath the sustentaculum tali, forcing medial translation to centralize the tuberosity under the tibial load axis. A Steinmann wire or Schanz pin serves as a joystick to guide the tuberosity posteriorly and plantarly, increasing calcaneal length and height. Coronal rotation is also corrected during this process, reducing varus alignment.

Once fragment reduction is complete, the lateral wall is pushed medially to correct widening. Temporary fixation with Kirschner wires is verified through fluoroscopy, followed by definitive fixation ( Fig. 3 ).

The ideal fixation strategy aims to achieve absolute stability of the subtalar and calcaneocuboid joints, using traction screws anchored to the sustentaculum tali. However, in cases with significant comminution, achieving absolute stability is challenging, requiring the use of positioning screws to maintain anatomical reduction.

The posterior tuberosity, lateral wall, and anterior portion of the calcaneus are fixed with relative stability, achieved through screws or plates, depending on the fracture's morphology and comminution.

Discussing synthesis options in these situations, Ni et al. 35 (2016) demonstrated that the mechanical stability of fractures fixed with locking plates is comparable to that achieved with screws alone. Subsequent studies confirmed similar rigidity, postoperative function, and rehabilitation outcomes between screws and plates. 25 36 37 Therefore, screw fixation is preferred in most cases due to less soft tissue envelope injury and lower cost.

**Fig. 4:**
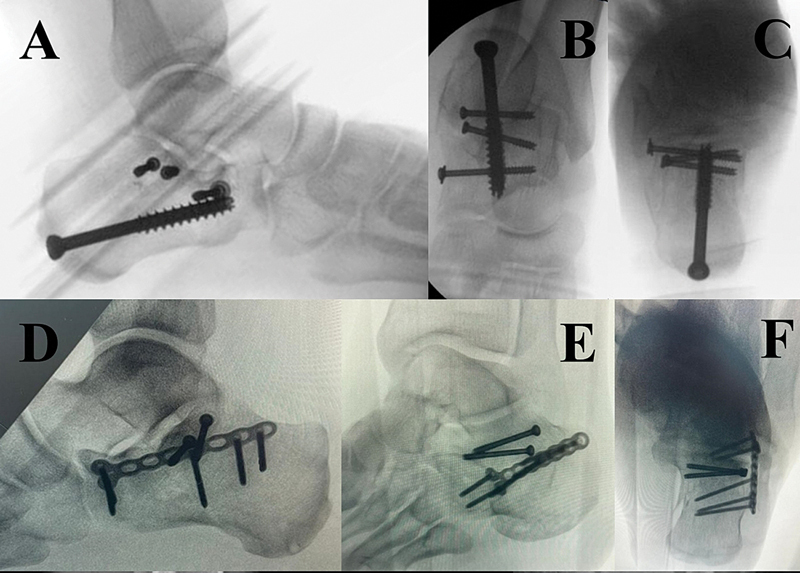
Screws and plate fixation. ( **A–C** ) Screws fixation reducing all the calcaneal fractures components. ( **D–F** ) Calcaneal anterior portion comminuted. Subtalar joint fixated with screws and calcaneal with locked plate.

Maintenance of the reduction of the tuberosity is achieved with a positioning screw, which is fixed to the anterior portion of the calcaneus. The screw can be directed from posterior to anterior or from dorsal and posterior to anterior and inferior, depending on the bone quality suitable for the screw to remain fixed 12 20 38 ( Fig. 4 ).

When comminution and lack of bone mass preclude stable screw fixation, locking plates are used to stabilize reductions between the anterior and posterior calcaneal elements. 2 Specific plates designed for the sinus tarsi approach or conventional calcaneal locking plates can be shaped for proper placement ( Fig. 4 ). The calcaneal nail is a current fixation option, following the same concepts described earlier, with encouraging initial results, providing stability comparable to that of the locked plate. However, more clinical studies are needed to understand in which situations its use adds the most benefits. 39

In cases of extensive joint and body comminution, in which fixation between fragments is unfeasible, even with plates, primary arthrodesis of the subtalar joint may be performed. This approach maintains calcaneal shape and hindfoot alignment. 6

## Extended Lateral Approach

Understanding the vascular anatomy is critical for planning surgical approaches effectively.

**Fig. 5:**
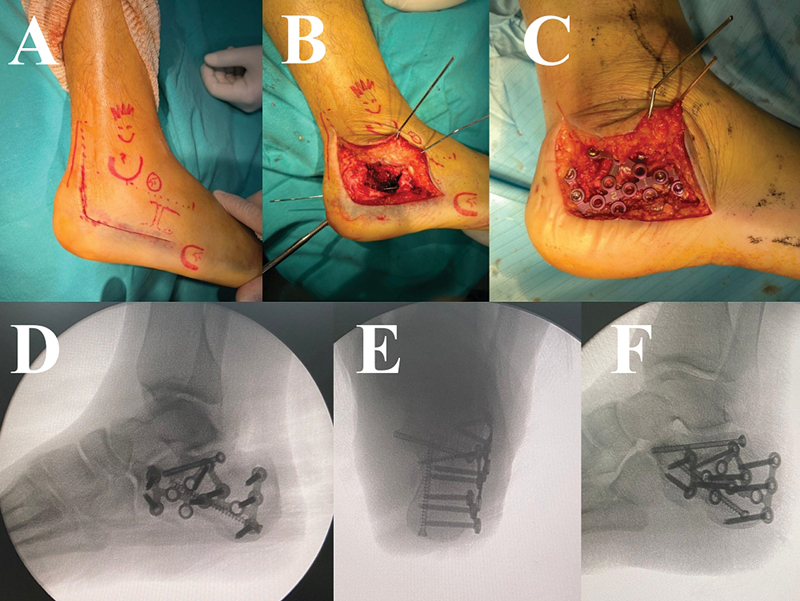
Extended Lateral Approach. ( **A** ) “L”-shaped lateral incision. ( **B** ) Schanz laterally for manipulation of tuberosity. ( **C** ) Calcaneal plate. ( **D** ) Lateral fluoroscopy view. ( **E** ) Axial fluoroscopy view. ( **F** ) Broden fluoroscopy view.

This approach, described by Benirschke 40 in 1993, positions the patient in a lateral decubitus position. An “L”-shaped lateral incision is used. ( Fig. 5A ) The deeper soft tissues are incised precisely along the skin incision and dissected together in a single plane down to the periosteum of the lateral wall. The vertical portion begins 2 cm proximally to the lateral malleolus tip, between the posterior third of the fibula and the anterior third of the Achilles tendon, with the sural nerve and lateral calcaneal artery located anteriorly. The horizontal portion between the dorsal and plantar skin, demarcated by compressing the heel, extending to the base of the fifth metatarsal. The two portions of the incision meet at an obtuse angle to minimize the risk of necrosis at the apex. 2

The flap is gently retracted during subperiorteal dissection along the lateral wall to the tip of the fibula. The entire flap is elevated as a single unit and maintained with two K-wires: one in the fibula and another in the talar neck. The flap is not manipulated again during the remainder of the procedure.

This approach provides 74% visualization of the subtalar joint (similar to the sinus tarsi approach), 71% of the lateral wall, and 3% of the anterior tuberosity. 27

The impacted superolateral articular fragment of the lateral wall is carefully elevated and placed in saline on the auxiliary table. Two K-wires are inserted from the posterior tuberosity without crossing the fracture line. Manipulation of the posterior tuberosity is performed using a 4.5-mm Schanz pin, placed laterally to medially, as described by Benirschke, 40 or posteriorly, as indicated by Rammelt and Zwipp 1 ( Fig. 5B ).

Manipulation follows a sequence: traction to restore length, medial translation, and then lateral translation to achieve physiological valgus. Once the correct position is obtained, the previously placed K-wires are advanced from the tuberosity into the sustentacular fragment, achieving temporary stabilization and restoration of the medial wall.

Subtalar joint reduction is performed from medial to lateral under direct vision, Broden projections, or dry arthroscopy. Compression is achieved with one or two screws. The tuberosity is aligned with the reduced joint, height and varus-valgus alignment are controlled, and fixation is completed with pins. The anterior process is reconstructed medially to laterally, using the cuboid as a guide. 2

Finally, the lateral wall is repositioned and fixed with a periarticular calcaneal plate, secured with screws in the posterior tuberosity, subtalar region, and anterior process. The plate aids in maintaining the calcaneal axis ( Fig. 5C ).

## Postoperative period

Postoperative care focuses on the evolution of the soft tissue envelope. A slightly compressive dressing is applied post-surgery to control bleeding. A removable orthosis supports early mobility while preventing equinus deformity.

After 10 to 14 days, sutures are removed, and efforts to improve range of motion and strength are intensified. At 10 weeks, progressive partial weight-bearing with the orthosis begins. By 12 weeks, with clinical and radiographic evidence of consolidation, full weight-bearing in firm-soled footwear is permitted. Radiographic follow-up is conducted periodically.

The moment to start weight bearing is still under debate, although most authors postpone it. Chongmuenwai and Thitirangsi 12 evaluated an earlier postoperative weight-bearing protocol. The authors compared a group that started progressive partial weight bearing as tolerated at 4 weeks postoperatively, with another group starting at 8 weeks. They observed no difference in maintenance of reduction between groups.

## Complications

Common complications of calcaneal fractures include posttraumatic osteoarthritis, neurological injuries, suture dehiscence, infections, nonunion, malunion, and fibular tendonitis. 4

## Conclusion

Calcaneal fractures significantly impact patients' lives, often leading to social and occupational challenges. A thorough understanding of the bone's anatomy and fracture patterns is critical for achieving optimal reduction. This process begins with the release of fracture fragments to allow precise anatomical positioning.

Surgical intervention via the sinus tarsi approach offers comparable functional outcomes to the extended lateral “L” approach while reducing soft tissue complications. Despite the longer learning curve, this approach should be strongly considered.

Articular and calcaneal body fixation can often be achieved with screws, provided an adequately sized constant fragment and sufficient bone density are present. Screws produce results comparable to those of locking plates, with soft tissue envelope injury and cost.

Locking plates are indicated when severe comminution precludes stable screw fixation. In cases of extensive joint and body comminution, primary subtalar joint arthrodesis can ensure calcaneal and hindfoot stability.

## References

[1] Rammelt S Zwipp H Calcaneus fractures: facts, controversies and recent developments Injury 2004 35 05 443 461 10.1016/j.injury.2003.10.006 15081321

[2] Rammelt S Swords M P Calcaneal Fractures-Which Approach for Which Fracture? Orthop Clin North Am 2021 52 04 433 450 10.1016/j.ocl.2021.05.012 34538353

[3] Sanders R Fortin P DiPasquale T Walling A Operative treatment in 120 displaced intraarticular calcaneal fractures. Results using a prognostic computed tomography scan classification Clin Orthop Relat Res 1993 290 87 95 8472475

[4] Epstein N Chandran S Chou L Current concepts review: intra-articular fractures of the calcaneus Foot Ankle Int 2012 33 01 79 86 10.3113/FAI.2012.0079 22381241

[5] Schepers T The sinus tarsi approach in displaced intra-articular calcaneal fractures: a systematic review Int Orthop 2011 35 05 697 703 10.1007/s00264-011-1223-9 21336854 PMC3080500

[6] Schepers T The primary arthrodesis for severely comminuted intra-articular fractures of the calcaneus: a systematic review Foot Ankle Surg 2012 18 02 84 88 10.1016/j.fas.2011.04.004 22443992

[7] Buckley R E Evidence for the best treatment for displaced intra-articular calcaneal fractures Acta Chir Orthop Traumatol Cech 2010 77 03 179 185 20619108

[8] Buckley R E Tough S Displaced intra-articular calcaneal fractures J Am Acad Orthop Surg 2004 12 03 172 178 10.5435/00124635-200405000-00005 15161170

[9] Sarrafian S K Kelikian A S Osteology Philadelphia Lippicott Williams & Wilkins 2011. p.40 119

[10] Guerado E Bertrand M L Cano J R Management of calcaneal fractures: what have we learnt over the years? Injury 2012 43 10 1640 1650 10.1016/j.injury.2012.05.011 22664393

[11] Wang Q Chen W Su Y Zhang Q Peng A Wu X Minimally invasive treatment of calcaneal fracture by percutaneous leverage, anatomical plate, and compression bolts–the clinical evaluation of cohort of 156 patients J Trauma 2010 69 06 1515 1522 10.1097/TA.0b013e3181e16150 21150529

[12] Chongmuenwai A Thitirangsi T Outcomes of Early Weight Bearing in Displaced Intra-articular Calcaneus Fractures Treated with Screws-Only Fixation Technique Indian J Orthop 2023 57 03 461 465 10.1007/s43465-023-00823-8 36825263 PMC9941380

[13] Schepers T Van Lieshout E M Van Ginhoven T M Heetveld M J Patka P Current concepts in the treatment of intra-articular calcaneal fractures: results of a nationwide survey Int Orthop 2008 32 05 711 715 10.1007/s00264-007-0385-y 17564705 PMC2551728

[14] Sanders R Intra-articular fractures of the calcaneus: present state of the art J Orthop Trauma 1992 6 02 252 265 10.1097/00005131-199206000-00022 1602349

[15] Sanders R Displaced intra-articular fractures of the calcaneus J Bone Joint Surg Am 2000 82 02 225 250 10.2106/00004623-200002000-00009 10682732

[16] Galluzzo M Greco F Pietragalla M De Renzis A Carbone M Zappia M Calcaneal fractures: radiological and CT evaluation and classification systems Acta Biomed 2018 89(1-S):138 150 10.23750/abm.v89i1-S.7017 29350643 PMC6179077

[17] Su Y Chen W Zhang T Wu X Wu Z Zhang Y Bohler's angle's role in assessing the injury severity and functional outcome of internal fixation for displaced intra-articular calcaneal fractures: a retrospective study BMC Surg 2013 13 40 10.1186/1471-2482-13-40 24330592 PMC3849198

[18] Baptista M Pinto R Torres J Radiological predictive factors for the outcome of surgically treated calcaneus fractures Acta Orthop Belg 2015 81 02 218 224 26280959

[19] Romash M M Calcaneal fractures: three-dimensional treatment Foot Ankle 1988 8 04 180 197 10.1177/107110078800800403 3350436

[20] Chongmuenwai A Wongfukiat O Choovongkomol K Postoperative 3D computed tomographic evaluation of 92 calcaneal fracture reduction using the sinus tarsi technique and fixation with 3.5 mm cortical screws Eur J Orthop Surg Traumatol 2024 34 06 2957 2962 10.1007/s00590-024-03998-4 38832997

[21] Yu S M Yu J S Calcaneal Avulsion Fractures: An Often Forgotten Diagnosis AJR Am J Roentgenol 2015 205 05 1061 1067 10.2214/AJR.14.14190 26496554

[22] Essex-Lopresti P The mechanism, reduction technique, and results in fractures of the os calcis Br J Surg 1952 39 157 395 419 10.1002/bjs.18003915704 14925322

[23] Sayyed-Hosseinian S H Shirazinia M Arabi H Aghaee M A Vahedi E Bagheri F Does the postoperative quality of reduction, regardless of the surgical method used in treating a calcaneal fracture, influence patients' functional outcomes? BMC Musculoskelet Disord 2023 24 01 562 10.1186/s12891-023-06697-z 37430205 PMC10331959

[24] Meena S Gangary S K Sharma P Review Article: Operative versus nonoperative treatment for displaced intraarticular calcaneal fracture: a meta-analysis of randomised controlled trials J Orthop Surg (Hong Kong) 2016 24 03 411 416 10.1177/1602400328 28031517

[25] Schepers T Sinus Tarsi Approach with Screws-Only Fixation for Displaced Intra-Articular Calcaneal Fractures Clin Podiatr Med Surg 2019 36 02 211 224 10.1016/j.cpm.2018.10.004 30784532

[26] Bakker B Halm J A Van Lieshout E MM Schepers T The fate of Böhler's angle in conservatively-treated displaced intra-articular calcaneal fractures Int Orthop 2012 36 12 2495 2499 10.1007/s00264-012-1706-3 23138968 PMC3508062

[27] Prather J Wilson J Abyar E Young S McGwin G Crocker C C Exposure of the Calcaneus in the Sinus Tarsi Approach Versus the Lateral Extensile Approach: A Cadaveric Study Foot Ankle Spec 2025 18(2):171 177 10.1177/19386400221114488 35880349

[28] Meng Q Wang Q Wu X Peng A Yan J Clinical application of the sinus tarsi approach in the treatment of intra-articular calcaneal fracture Medicine (Baltimore) 2018 97 13 e0175 10.1097/MD.0000000000010175 29595648 PMC5895366

[29] Zhang T Su Y Chen W Zhang Q Wu Z Zhang Y Displaced intra-articular calcaneal fractures treated in a minimally invasive fashion: longitudinal approach versus sinus tarsi approach J Bone Joint Surg Am 2014 96 04 302 309 10.2106/JBJS.L.01215 24553886

[30] Yao H Liang T Xu Y Hou G Lv L Zhang J Sinus tarsi approach versus extensile lateral approach for displaced intra-articular calcaneal fracture: a meta-analysis of current evidence base J Orthop Surg Res 2017 12 01 43 10.1186/s13018-017-0545-8 28288661 PMC5348794

[31] Kikuchi C Charlton T P Thordarson D B Limited sinus tarsi approach for intra-articular calcaneus fractures Foot Ankle Int 2013 34 12 1689 1694 10.1177/1071100713510267 24151194

[32] Guo C Xu Y Li C Li X Wang Z Cai M Xu X Comparing less invasive plate fixation versus screw fixation of displaced intra-articular calcaneus fracture via sinus tarsi approach Int Orthop 2021 45 09 2231 2237 10.1007/s00264-020-04867-5 33145609

[33] Nosewicz T Knupp M Barg A Maas M Bolliger L Goslings J C Hintermann B Mini-open sinus tarsi approach with percutaneous screw fixation of displaced calcaneal fractures: a prospective computed tomography-based study Foot Ankle Int 2012 33 11 925 933 10.3113/FAI.2012.0925 23131437

[34] Mao J T Chen C M Lin C W Lu H L Kuo C C Comparison of the Radiographic and Clinical Outcomes between the Sinus Tarsi and Extended Lateral Approaches for Intra-Articular Calcaneal Fractures: A Retrospective Study J Pers Med 2024 14 03 259 10.3390/jpm14030259 38541001 PMC10970954

[35] Ni M Wong D WC Mei J Niu W Zhang M Biomechanical comparison of locking plate and crossing metallic and absorbable screws fixations for intra-articular calcaneal fractures Sci China Life Sci 2016 59 09 958 964 10.1007/s11427-016-0010-9 27349998

[36] Ni M Mei J Li K Niu W Zhang M The primary stability of different implants for intra-articular calcaneal fractures: an in vitro study Biomed Eng Online 2018 17 01 50 10.1186/s12938-018-0484-6 29716591 PMC5930824

[37] Fan B Zhou X Wei Z Ren Y Lin W Hao Y Cannulated screw fixation and plate fixation for displaced intra-articular calcaneus fracture: A meta-analysis of randomized controlled trials Int J Surg 2016 34 64 72 10.1016/j.ijsu.2016.08.234 27565242

[38] Wang Q Zhang N Guo W Wang W Zhang Q Cannulated screw fixation versus plate fixation in treating displaced intra-articular calcaneus fractures: a systematic review and meta-analysis Int Orthop 2021 45 09 2411 2421 10.1007/s00264-021-05141-y 34370059

[39] Liu B Ma L Liu C Zhang B Wu G [A prospective study on treatment of Sanders type II and III calcaneal fractures with interlocking intramedullary nail fixation system] Zhongguo Xiu Fu Chong Jian Wai Ke Za Zhi 2024 38 03 303 308 10.7507/1002-1892.202312076 38500423 PMC10982044

[40] Benirschke S K Sangeorzan B J Extensive intraarticular fractures of the foot. Surgical management of calcaneal fractures Clin Orthop Relat Res 1993 292 128 134 8519099

